# Complete mitochondrial genome of the crinoid *Poliometra prolixa* (Crinoidea: Comatulida: Antedonidae)

**DOI:** 10.1080/23802359.2023.2252129

**Published:** 2023-09-04

**Authors:** Hayoung Kwon, Hyoung Sook Park, Jae-Sung Rhee

**Affiliations:** a Department of Marine Science, College of Natural Sciences, Incheon National University, Incheon, South Korea; b Department of Song-Do Biological Engineering, Incheon Jaeneung University, Incheon, South Korea; c Yellow Sea Research Institute, Incheon, South Korea

**Keywords:** Complete mitogenome, Crinoidea, *Poliometra prolixa*, phylogenetic analysis

## Abstract

*Poliometra prolixa* Sladen, 1881, is a comatulid crinoid found in the Arctic deep sea. In this study, we report the complete mitochondrial genome sequence of *P. prolixa* (Comatulida: Antedonidae). The complete mitogenome of *P. prolixa* was 15,916 bp long and comprised 13 protein-coding genes (PCGs), 22 transfer RNA genes, and 2 ribosomal RNA genes. The base composition of the *P. prolixa* mitogenome was 24.0% A, 44.9% T, 19.0% G, and 12.1% C. Phylogenetic analysis using all PCGs of the complete mitogenome confirmed the inclusion of *P. prolixa* within the Comatulida.

## Introduction

The phylum Echinodermata Klein, 1778, exclusively includes marine animals. Echinoderms belong to the phylum Echinodermata and comprise five distinct classes: Asteroidea de Blainville, 1830; Crinoidea Miller, 1821; Echinoidea Schumacher, 1817; Holothuroidea de Blainville, 1834; and Ophiuroidea Gray, 1840. The class Crinoidea is a diverse clade of living echinoderms with substantial fossil records spanning approximately 500 million years (Hess et al. 1999). Among the extant classes of echinoderms, crinoids are the least studied, with approximately 600 extant and 8,000 fossil species (Kroh and Smith 2010; Rouse et al. 2013). Given their morphometry, diversity, ecological dynamics, complex geological history, and evolutionary radiation, taxonomic analyses have been conducted to understand the evolutionary heritage of crinoids (Ausich et al. 2015; Cole 2017; Wright et al. 2017). Concerted efforts with genomic information on crinoids have been made to reveal their phylogenetic relationships and nomenclatural stability, and some results have provided taxonomic platforms and systematic revisions of extant crinoids (Foote 1999; Janies 2001; Telford et al. 2014; Wright et al. 2017). However, crinoid genomic resources are insufficient to complement their robust taxonomic status at the molecular level.

The genus *Poliometra* consists of a single species, *Poliometra prolixa* Sladen, 1881, which is a comatulid crinoid species. *Poliometra prolixa* is prominently distributed in the Arctic deep sea; however, detailed observations have been recorded from samples collected from the Beaufort Sea (Miller 2012) and eastern Fram Strait, west of Svalbard (Meyer et al. 2014). Exploration of phylogenetic relationships and genetic distances in *P. prolixa* has been limited because of a lack of morphometric details and habitat information and insufficient genomic data. To address this knowledge gap, it is crucial to obtain comprehensive information on the *P. prolixa* mitochondrial genome. Such information can serve as a valuable reference for understanding the phylogenetic distances and relationships between *P. prolixa* and other crinoids, providing insights into gene flow and evolutionary processes at high resolution. Partial marker genes, including *16S* ribosomal RNA (*rRNA*; GenBank accession no. KC626669), *18S rRNA* (KC626763), *28S rRNA* (KC626857), and *cox1* (KC626577), have been reported in *P. prolixa*; however, a complete mitogenome sequence for this species is not yet available. To this end, we aimed to sequence and characterize the entire mitogenome of *P. prolixa* to provide a complete mitogenome reference that is valuable for determining robust phylogenetic relationships and population genomics in crinoids. The complete mitogenome may serve as a valuable resource for robust phylogenetic analyses and population genomics of crinoids.

## Materials and methods

**Figure 1.**
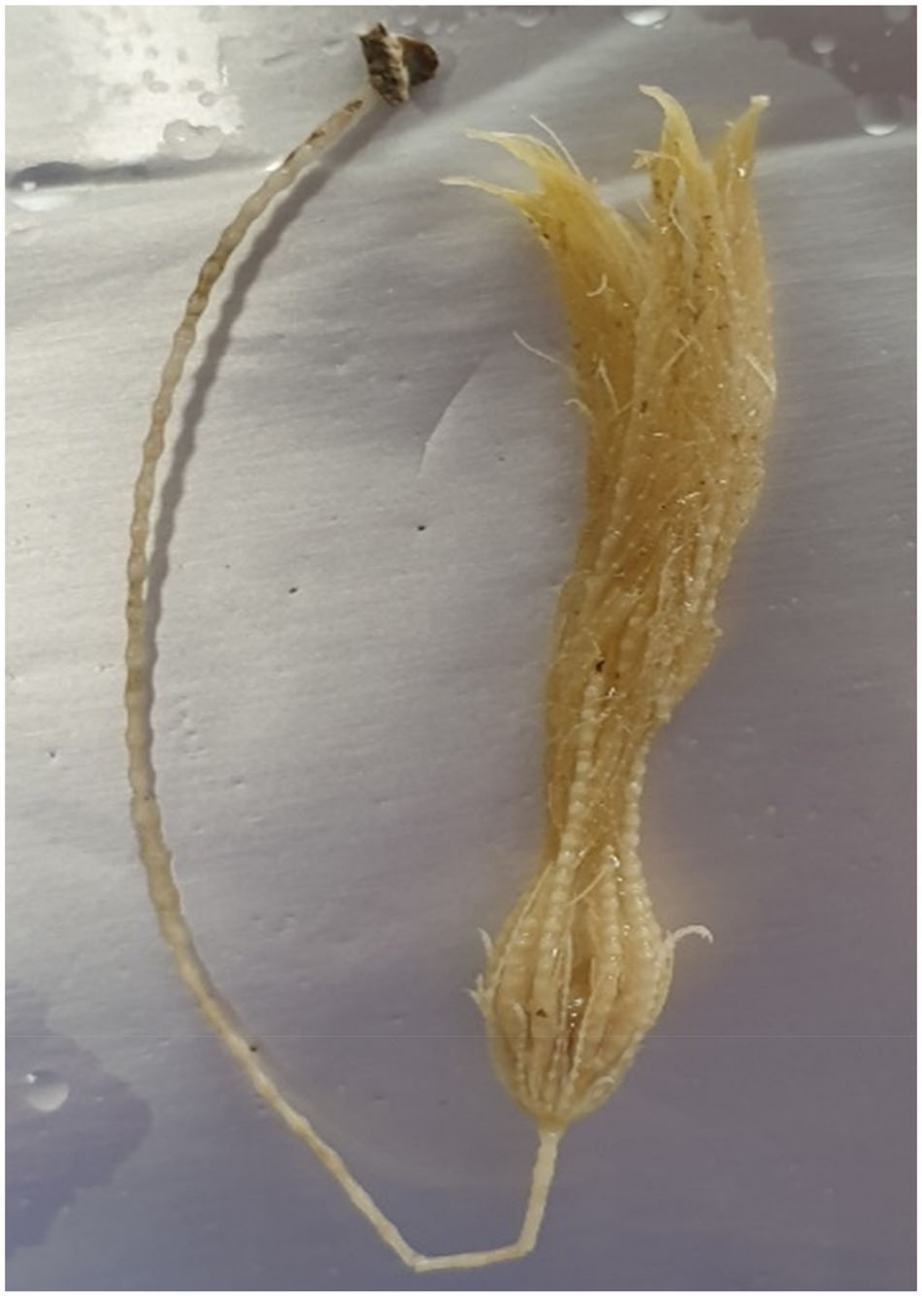
Species reference image of *Poliometra prolixa* collected from the Beaufort Sea.

An individual specimen of *P. prolixa* (Figure 1) was sampled from the Beaufort Sea (69°52′N, 139°03′W) with a remotely operated underwater vehicle (Monterey Bay Aquarium Research Institute). Partial tissues of *P. prolixa* after DNA extraction were registered in the collection of the Research Institute of Basic Sciences of Incheon National University, Incheon, South Korea (Dr. Sang-Eun Nam: se_nam2@inu.ac.kr) under Tissue No. Echinodermata-45. Total genomic DNA was isolated from partial tissue of *P. prolixa* using the DNeasy Blood and Tissue Kit (Qiagen, Hilden, Germany) according to the manufacturer’s instructions. Next-generation sequencing was performed to obtain a circular mitogenome with the HiSeq platform (150 bp; HiSeq X ten; Illumina, San Diego, CA, USA), using our previously described protocols (Nam and Rhee 2020). Prior to Illumina HiSeq sequencing, a fragment library was constructed using a TruSeq DNA Sample Preparation Kit (Illumina, San Diego, CA, USA) according to the manufacturer’s protocol (Macrogen, Inc., Seoul, South Korea). For sequencing library preparation, purified DNA samples were sheared *via* random fragmentation, and then 5′ and 3′ adapters were ligated. The library was sequenced using the Illumina HiSeq platform and paired-end raw reads were quality controlled using FastQC version 0.11.9 (Andrews 2010). Raw reads were demultiplexed, and matched index pairs alone were retained for further processing. The raw read data were quality-trimmed to remove adapter sequences, low-quality reads, reads with >10% unknown bases, and ambiguous bases using Trimmomatic (Bolger et al. 2014), resulting in a high-quality assembly. From 5,725,692,292 raw reads, 37,918,492 filtered reads were obtained. *De novo* assembly was conducted to obtain a circular contig of the *P. prolixa* mitogenome with various k-mers using SPAdes (Bankevich et al. 2012). The average depth of coverage is shown in Supplementary Figure 1. The resulting contig consensus sequence was annotated using MITOS2 (Bernt et al. 2013) and tRNAscan-SE 2.0 (Lowe and Eddy 1997). A mitochondrial genome map of *P. prolixa* was drawn using the OGDRAW web server (https://chlorobox.mpimp-golm.mpg.de/OGDraw.html).

To assess the phylogenetic relationships of *P. prolixa*, a maximum-likelihood phylogenetic tree was constructed using all the amino acid coding sequence regions concatenated from *P. prolixa* mitogenome and 33 other echinoderms (13 asteroids, 6 crinoids, 3 echinoids, 4 holothuroids, and 6 ophiuroids). The sequences were aligned using ClustalX 2.0 (Larkin et al. 2007). To determine the best substitution model, the JModelTest ver. 2.1.10 (Darriba et al. 2012) was employed, and the GTR + G + I model was applied to perform a maximum-likelihood analysis using PhyML 2.4.5 (Guindon and Gascuel 2003). The percent confidence values for each node were calculated from 1000 bootstrap replicates.

## Results

**Figure 2.**
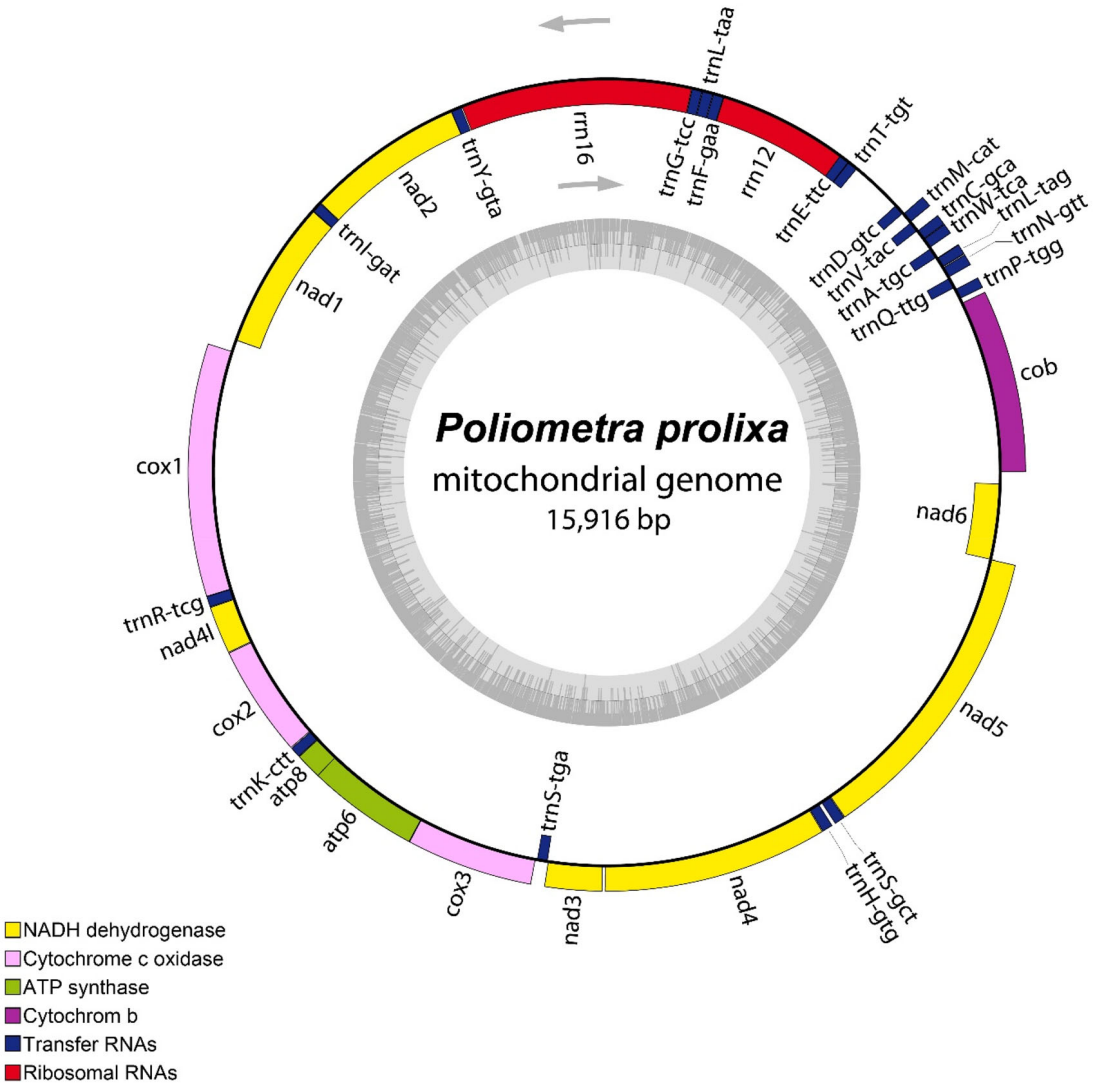
Circular map of the assembled *P. prolixa* mitochondrial genome, consisting of 13 protein-coding, 22 transfer RNA, and two ribosomal RNA genes. Genes encoded on the reverse strand and forward strand are illustrated inside and outside the circles, respectively. This map was drawn using the OGDRAW web server (https://chlorobox.mpimp-golm.mpg.de/OGDraw.html).

The complete mitogenome of *P. prolixa* was found to be 15,916 bp long (GenBank accession no. OP177937) and contained 13 protein-coding genes (PCGs), two rRNA genes (12S and 16S), and 22 transfer RNA (tRNA) genes (Figure 2). The most commonly shared start codon for the 13 PCGs was ATG. The termination codons used were TAA (*nad1*, *cox1*, *nad4l*, *atp6*, *nad4*, *nad5*, and *nad6*) and TAG (*cob*, *nad2*, *cox2*, *atp8*, *cox3*, and *nad3*). The nucleotide sequence of *cox1* exhibited the highest similarity (99.9%) to that of *cox1* sequence of *P. prolixa* previously registered in GenBank (KC626577). The overall nucleotide composition of the mitogenome showed a GC content of 31.1% and 24.0% A, 44.9% T, 19.0% G, and 12.1% C.

**Figure 3.**
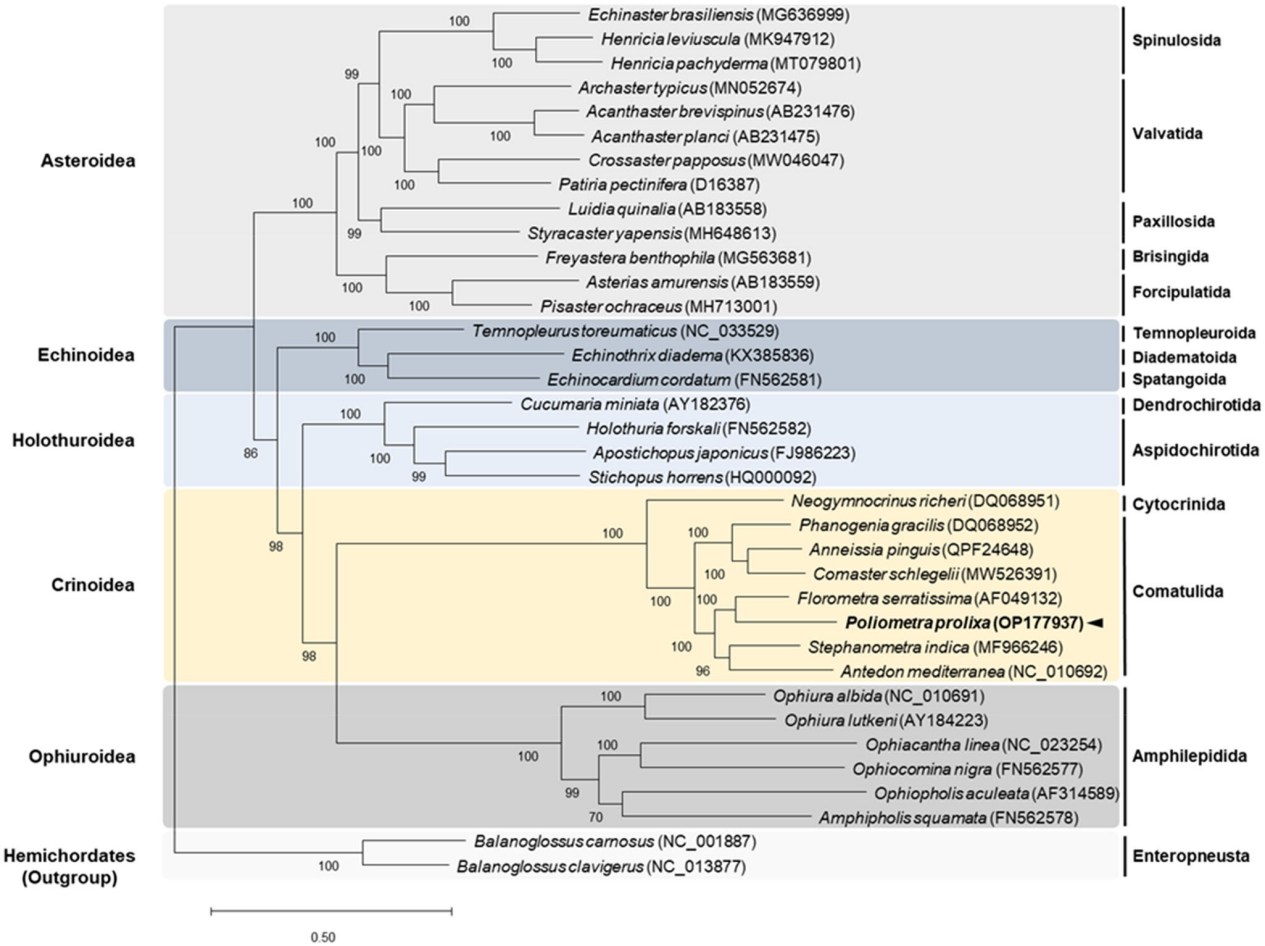
Maximum-Likelihood (ML) phylogenetic tree of 33 published complete Mitogenomes of Echinodermata, including that of *P. prolixa*, based on the concatenated nucleotide sequences of protein-coding genes (PCGs). The numbers on the branches indicate ML bootstrap percentages. DDBJ/EMBL/genbank accession numbers for published sequences are incorporated. The black arrow represents the sea pen analyzed in this study.

Phylogenetic analysis of the protein-coding genes confirmed that *P. prolixa* belongs to the family of comatulids (Figure 3). *Poliometra prolixa* is closely related to *Florometra serratissima* Clark, 1907, a member of the family Antedonidae, and grouped with *Antedon mediterranea* Lamarck, 1816 and *Stephanometra indica* Smith, 1876.

## Discussion and conclusions

Here, we present the first complete mitochondrial genome for *P. prolixa*. Providing the genetic reference for *P. prolixa* is essential as this species is a single member of the genus *Poliometra. P. prolixa* is a geographically representative crinoid, with distributions reported in the Beaufort Sea and Fram Strait (Miller 2012; Meyer et al. 2014). Of the four major taxa in extant crinoids, Comatulida comprises approximately 83% of all extant crinoids (Hess and Messing 2011). Comatulida is the most diversified order of extant crinoids; however, its classification is still primarily based on morphological characteristics, and its ontogeny remains controversial owing to the use of highly plastic diagnostic taxonomic characters and the lack of genomic information. Researchers have begun to incorporate genomic data for phylogenetic construction in Comatulida (Hemery et al. 2013; Rouse et al. 2013; Summers et al. 2014; Taylor et al. 2017); however, taxonomy has been constantly revised at the family level and overall comatulid ontogeny (Hemery et al. 2013; Naughton et al. 2014; Summers et al. 2017; Taylor et al. 2018). Considering that the family Antedonidae is the most speciose among the Comatulida with a broad scattering of genera, the complete mitochondrial genome of *P. prolixa* reported in this study contributes to the understanding of the evolutionary relationships of this species within the comatulid crinoids. Additionally, this study provides a reference for future investigations on molecular-based species identification, population structure, and genetic diversity of *P. prolixa* across its geographic range and evolutionary history.

## Supplementary Material

Supplemental Material

## Data Availability

BioProject, BioSample, and SRA accession numbers are https://www.ncbi.nlm.nih.gov/bioproject/PRJNA916601, https://www.ncbi.nlm.nih.gov/biosample/?term=SAMN32487547, and https://www.ncbi.nlm.nih.gov/sra/SRR22923304, respectively. The data that support the findings of this study are openly available in the National Center for Biotechnology Information (NCBI) at https://www.ncbi.nlm.nih.gov, with an accession number OP177937.
