# Supplementary figures and images for: Complete mitochondrial genome of the crinoid Poliometra prolixa (Crinoidea: Comatulida: Antedonidae)
Source: Mitochondrial DNA B Resour. 2023 Sep 4;8(9):927–31. doi: 10.1080/23802359.2023.2252129 (PMC10478607; doi:10.1080/23802359.2023.2252129)

Supplementary figure 1. The coverage depth of mitochondrial genome of *Poliometra prolixa*.


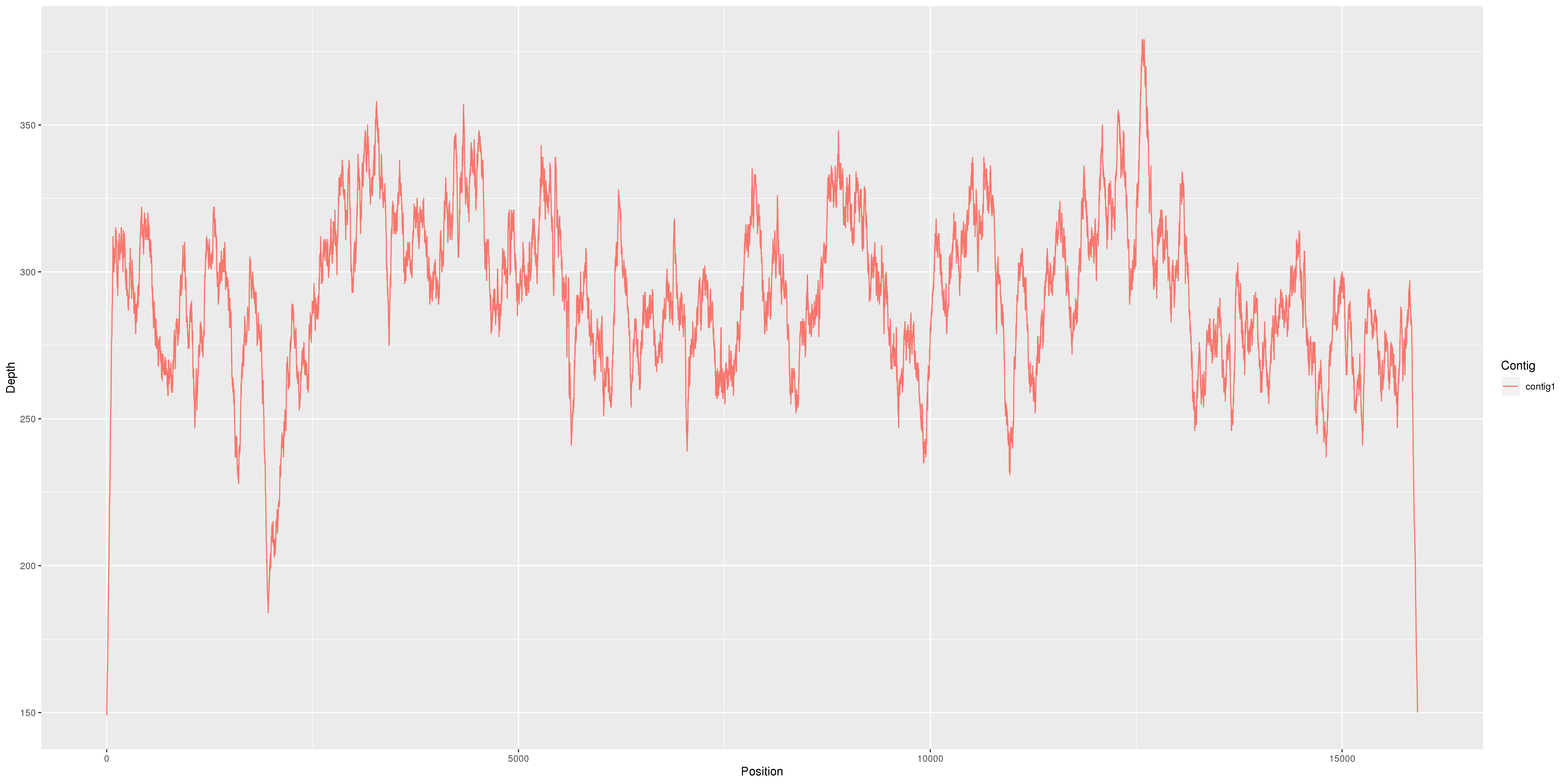

Supplement: Supplemental Material [file TMDN_A_2252129_SM2192.docx]
